# Acute ischemic stroke due to unruptured small aneurysm of internal carotid artery: A case report

**DOI:** 10.1097/MD.0000000000022656

**Published:** 2020-10-09

**Authors:** Hongjun Su, Na Zhao, Kun Zhao, Xuejuan Zhang, Riguang Zhao

**Affiliations:** a Department of Neurology; b Department of General Medicine, Tianjin Baodi Hospital, Bao Di Clinical College of Tianjin Medical University, Baodi, Tianjin, China.

**Keywords:** acute ischemic stroke, aneurysm, digital subtraction angiography, internal carotid artery

## Abstract

**Rationale:**

Intracranial small aneurysm is a rare cause of ischemic stroke, and been described only in sparse case reports. The exact pathophysiology, treatment strategies, and prognosis remain incompletely understood.

**Patient concerns:**

A 42-year-old man presented with an acute onset weakness of the right limbs.

**Diagnoses:**

Neuroimaging evaluation confirmed a diagnosis of acute ischemic stroke and left internal carotid artery (ICA) small aneurysm.

**Interventions:**

The patient underwent oral anti-platelet therapy (100 mg aspirin daily).

**Outcomes:**

The patient recovered to normal status within 4 weeks following antiplatelet treatment. During a follow-up period of 1 year, he remained neurologically asymptomatic and led a virtually normal life.

**Lessons:**

It is crucial for clinicians to be aware of this entity, as cerebral infarction caused by small cerebral aneurysm is extremely rare.

## 1 Introduction

Unruptured aneurysms are a rare cause of cerebral infarction or transient ischemic attack (TIA). A epidemiological studies showed the incidence of ischemic cerebrovascular disease in unruptured aneurysms is 3.3%, the average size of aneurysms is 12.5 mm (5–45 mm), and most of them are located in the anterior circulation.^[1]^ Intra-aneurysmal thrombosis is the mechanism of cerebral ischemia in unruptured aneurysms. Treatment of unruptured cerebral aneurysm causing brain infarction is controversial. Some cases have been treated surgically, whereas others were treated conservatively with or without medication.^[2]^ Herein, we report a case of acute cerebral infarction caused by internal carotid artery small aneurysm in which effective antiplatelet therapy led to rapid recovery.

### 1.1 Ethics statement

This study was approved by review board of Tianjin Baodi hospital. Informed consent for publication of this report was obtained from the patient.

## 2 Case report

**Figure 1:**
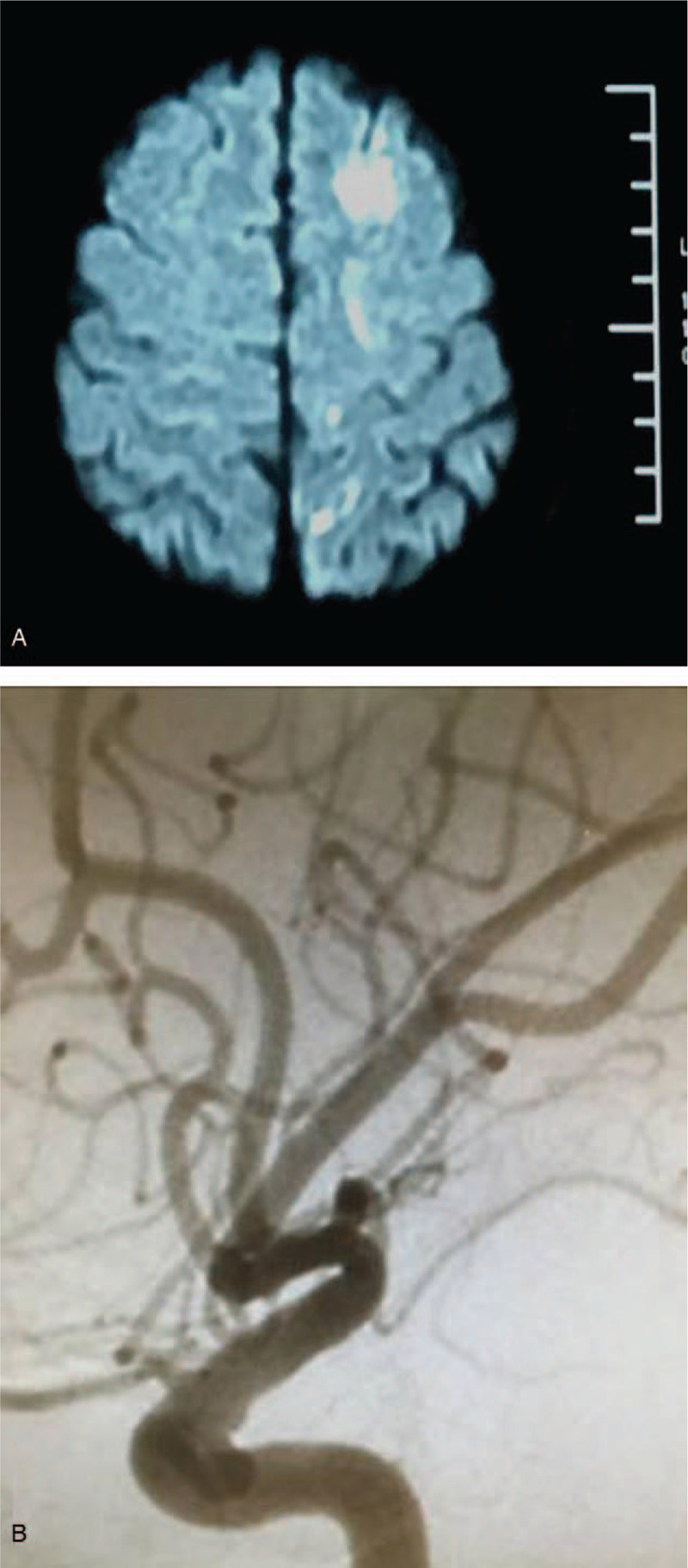
Diffusion-weighted magnetic resonance imaging (MRI-DWI), digital subtraction angiography (DSA). (A) MRI-DWI showed acute ischemic stroke in the left frontal and parietal lobe. (B) DSA showed unruptured intracranial aneurysm on supraclinoidal segments of left internal carotid artery.

A 42-year-old man presented to the emergency department with 3-day course of weakness of right limbs. He also complained of a little bit speech blur. His medical history was normal. He had no history of hypertension or diabetes. Review of systems was negative for headache. He does not smoke cigarette and drink alcohol. He was no familial history of aneurysm. Physical examination showed incomplete motor aphasia, right facial nerve paralysis, weakness of the right limbs, and a positive pathologic reflex. The muscle strengths of the right extremity were grade 4. The patient's neurological function was graded as 2 points according to the National Institutes of Health Stroke Scale (NIHSS) and 1 point according to the modified Rankin Scale (mRS). A magnetic resonance imaging (MRI) scan of the brain showed acute cerebral infarction at left frontal-parietal lobe. Brain diffusion-weighted magnetic resonance imaging (MRI-DWI) showed a focal area of restricted diffusion within the left frontal and parietal lobe (Fig. 1A). He was diagnosed with an acute ischemic stroke. The patient was subsequently transferred to neurology department and was given an oral anti-platelet therapy (100 mg aspirin daily).

Laboratory data were within normal limits, including a complete blood count, coagulation function, hemorrheology, liver function, fasting blood glucose, blood lipid, anti-cardiolipin antibody. Anti-treponema pallidum and human immunodeficiency virus (HIV) antibody were negative. A urine drug screen was negative. No other sources of embolism were found on transthoracic echocardiogram and Holter monitoring. There were no other lesion and no predisposing risk factors that produced cerebral ischemia. Further brain digital subtraction angiography (DSA) confirmed the saccular aneurysm (3 mm × 4.5 mm) in the supraclinoidal segments of left internal carotid artery (Fig. 1B). DSA ruled out carotid atherosclerotic disease. It was thought that the aneurysm was the source of emboli resulting in left frontal-parietal infarction. An oral antiplatelet regimen was scheduled (100 mg aspirin daily), and he was discharged home on hospital day 7. He was followed up at the neurology department as an outpatient. One month later, the patient's symptoms were completely relieved without any notable neurological sequelae. The NIHSS and mRS scores were both improved to 0 point. The patient did not have any procedures to treat the aneurysms. During a follow-up period of 1 year, he remained free of recurrent stroke, and the aneurysms remained unchanged on computed tomography angiography (CTA).

## 3 Discussion

Unruptured intracranial aneurysm (UIA) is generally asymptomatic and detected incidentally, but symptoms of ischemic are occasionally observed. Cerebral infarction caused by small cerebral aneurysm is extremely rare, ischemic symptoms were observed in 3% to 3.6% of unruptured aneurysm.^[1]^ Formation of thrombus was detected in 48% aneurysms of 20 to 25 mm diameter and in 76% of aneurysms of over 25 mm diameter. Partially thrombosed cerebral aneurysm is associated with thromboembolic events due to propagation of mural thrombus.^[3–5]^ Intramural thrombi are thought to be one of the causative factor of ischemic symptoms. Factors associated with UIA thrombosis include the ratio between aneurysmal volume and aneurysmal neck size, the age of the aneurysm, and intrasaccular hemodynamic changes that involve endothelial damage. Cerebral infarction due to distal embolization, local extension of the luminal thrombus, and increased mass effect. Reduce of blood inflow, increased blood viscosity, and turbulent blood flow are important causative factors in the formation of intramural thrombi. The endothelium of the aneurysm wall are damaged by turbulent blood flow in aneurysm dome and cause exposure of subendothelial matrix, and induce platelet aggregation. Thrombotic emboli from the thrombosed aneurysm caused occlusion of the branches.^[6]^

This paper presents a patient with acute ischemic stroke caused by an UIA. In this case, the imaging features of the infarcts were suggestive of embolism, alternative etiologies were ruled out. These features suggest a causal relationship between the aneurysm and the infarct. By far, there is controversy and no consensus about the best way to approach patients with UIA and ischemic stroke.^[7–9]^ In the present case, the cerebral aneurysm was first detected at the acute stage of brain infarction and conservative therapy was instituted. However, the possibility of aneurysm rupture should be considered if carotid angiography shows rapid resolution of the intramural thrombus.

Antiplatelets may inhibit platelet aggregation and activation within the aneurysmal sac and reduce the risk of ischemic events.^[10]^ The risk of subarachnoid hemorrhage while on antiplatelet medication is unknown. Our patient received a low dose of aspirin, and we did not observe hemorrhagic complications. He does not have recurrent ischemic stroke at the time of follow-up. The role of surgery or endovascular interventions in patients with UIA and ischemic stroke, however, is not clear. Zhang et al's study showed that invasive surgical approach, intraoperative monitoring, effective management of cerebrovascular perfusion, and coagulation can prevent the development of cerebral ischemia and aneurysm rupture.^[11]^ However, manipulation of the intrasaccular thrombus during coiling can lead to further embolization,^[12]^ given the risk of intraoperative thromboembolic events, it is possible that the risk of surgery in these patients is higher than in patients with asymptomatic UIA. Further studies are needed before this option can be widely implemented in these patients.

In conclusion, an unruptured small aneurysm of left internal carotid artery is verified in the present case. It is crucial for all physicians and paramedics to be aware of this entity as the etiology is quite challenging. Antiplatelet therapy may be effective in some patients, and the prognosis can be favorable.

## Acknowledgments

The authors would like to thank the patient for his participation in this study.

## Author contributions

**Conceptualization:** Riguang Zhao.

**Investigation:** Hongjun Su, Na Zhao, Kun Zhao, Xuejuan Zhang.

**Writing – original draft:** Hongjun Su.

**Writing – review & editing:** Riguang Zhao.

## Abbreviations

Abbreviations: DSA = digital subtraction angiography, HIV = human immunodeficiency virus, ICA = internal carotid artery, MRI = magnetic resonance imaging, MRI-DWI = diffusion-weighted magnetic resonance imaging, mRS = modified Rankin Scale, NIHSS = National Institutes of Health Stroke Scale, TIA = transient ischemic attack, UIA = unruptured intracranial aneurysm.
